# Cell layer-specific patterns of cell division and cell expansion during fruit set and fruit growth in tomato pericarp

**DOI:** 10.1093/jxb/erx058

**Published:** 2017-03-28

**Authors:** Jean-Pierre Renaudin, Cynthia Deluche, Catherine Cheniclet, Christian Chevalier, Nathalie Frangne

**Affiliations:** 1 UMR 1332 BFP, INRA National Institute for Agronomic Research, University of Bordeaux, F-33882 Villenave d’Ornon Cedex, France; 2 UMS 3420, Bordeaux Imaging Center, CNRS, US4, INSERM, University of Bordeaux, F-33000 Bordeaux, France

**Keywords:** Cell division, cell expansion, endoreduplication, fruit, fruit set, growth rate, pericarp, *Solanum lycopersicum*, tomato.

## Abstract

In angiosperms, the ovary wall resumes growth after pollination through a balanced combination of cell division and cell expansion. The quantitative pattern of these events remains poorly known in fleshy fruits such as tomato (*Solanum* spp.), in which dramatic growth of the pericarp occurs together with endoreduplication. Here, this pattern is reported at the level of each of the cell layers or groups of cell layers composing the pericarp, except for vascular bundles. Overall, cell division and cell expansion occurred at similar rates for 9 days post anthesis (DPA), with very specific patterns according to the layers. Subsequently, only cell expansion continued for up to 3–4 more weeks. New cell layers in the pericarp originated from periclinal cell divisions in the two sub-epidermal cell layers. The shortest doubling times for cell number and for cell volume were both detected early, at 4 DPA, in epicarp and mesocarp respectively, and were both found to be close to 14 h. Endoreduplication started before anthesis in pericarp and was stimulated at fruit set. It is proposed that cell division, endoreduplication, and cell expansion are triggered simultaneously in specific cell layers by the same signals issuing from pollination and fertilization, which contribute to the fastest relative fruit growth early after fruit set.

## Highlight

Cell expansion and cell division resume simultaneously at fruit set in specific cell layers of the tomato pericarp, and display complementary quantitative importance during fruit growth.

## Introduction

Fruit growth, the longest phase in fruit development, is initiated by signals related to pollination and to fertilization (McAtee *et al.*, 2013; Sotelo-Silveira *et al.*, 2014). These processes trigger fruit set and rapid fruit growth through cell division and cell expansion, whose importance impacts fruit quality according to genotypes and environmental conditions (Nitsch, 1965; Coombe, 1976; Gillaspy *et al.*, 1993; Seymour *et al.*, 2013).

The molecular regulation of fruit set and growth has been intensively studied in various model species, including *Arabidopsis thaliana* and tomato (*Solanum* spp.) (Tanksley, 2004; Chevalier *et al.*, 2011; McAtee *et al.*, 2013; Seymour *et al.*, 2013; Karlova *et al.*, 2014). The organization of the tomato berry, a major model of fleshy fruits, has long been the focus of anatomical and cytological studies (Varga and Bruinsma, 1976; Roth, 1977; Gillaspy *et al.*, 1993; Tanksley, 2004; Cheniclet *et al.*, 2005; Xiao *et al.*, 2009; Pabón-Mora and Litt, 2011). Tomato fruit comprises pericarp and septa, derived from carpel walls; the placenta in a central position; and a locular tissue which differentiates from placenta and embeds seeds during fruit growth. In pericarp, a phase of cell divisions occurs for 7–20 days post anthesis (DPA) according to genotype and environment, mostly in the outer pericarp (Tanksley, 2004; Xiao *et al.*, 2009; Pabón-Mora and Litt, 2011). It contributes notably to a 1.5- to 3-fold increase in the number of cell layers within the pericarp (Mazzucato *et al.*, 1998; Cong *et al.*, 2002; Cheniclet *et al.*, 2005; Fanwoua *et al.*, 2012). Cell expansion has been reported to start between 2 and 8 DPA in mesocarp tomato cells (Cheniclet *et al.*, 2005; Xiao *et al.*, 2009; Pabón-Mora and Litt, 2011).

Both the quantitative importance of cell division and expansion in pericarp and the spatial and dynamic patterns of these two phenomena during fruit growth remain poorly described, as pointed out more than 20 years ago by Gillaspy *et al.* (1993). This may be driven by the large diversity of tomato fruit phenotypes and by the difficulty in quantifying these phenomena in developing fruits. In addition, there is looseness in the naming of the different groups of cell layers within the pericarp (Pabón-Mora and Litt, 2011). According to authors, exocarp and endocarp may relate to the single outer and inner epidermal layers, respectively, or may comprise rows of hypodermal tissues just beneath. In the same way, the mesocarp may include all cell layers except the two epidermal layers, or only those external to vascular bundles. Moreover, it is not clear what, if any, biological, evolutionary, or functional meaning these terms may have (Pabón-Mora and Litt, 2011).

Most of tomato fruit cells display highly endoreduplicated nuclei (Bergervoet *et al.*, 1996; Joubès *et al.*, 1999, Cheniclet *et al.*, 2005). Endoreduplication comes from the endocycle, a modified cell cycle (de Veylder *et al.*, 2011; Chevalier *et al.*, 2011). The functional role of endoreduplication in cell expansion is still a matter of debate (Sugimoto-Shirasu and Roberts, 2003; De Veylder *et al.*, 2011). Endoreduplication has been correlated with different fruit traits such as fruit size (Cheniclet *et al.*, 2005; Nafati *et al.*, 2011) and the duration of fruit growth (Chevalier *et al.*, 2011; Pirrello *et al.*, 2014). Despite the occurrence of a specific pattern of endoreduplication within the pericarp (Bourdon *et al.*, 2011), the functional relationship of this phenomenon with cell division and cell expansion remains unknown.

We address here the temporal and spatial quantitative pattern of cell division and of cell expansion within tomato pericarp during fruit growth. By using a new, cell layer-based approach, we identified that the earliest cellular events occurring at fruit set include mitotic induction, endoreduplication, and cell expansion in specific cell layers. The patterning of new cell layers after anthesis is also described, and a coherent naming for pericarp areas is proposed. These results provide a detailed framework for understanding fruit set and pericarp development.

## Materials and methods

### Plant material

Cherry tomato plants (*Solanum lycopersicum*), line West Virginia 106 (Wva106), were cultivated in a greenhouse as previously described (Cheniclet *et al.*, 2005). Flowers and fruit were collected at various stages at positions two to five in the first to seventh trusses for cytology and ploidy analyses. Two time-course experiments were conducted to study cellular patterns in pericarp according to time. Experiment 1, performed in 2004 and already partly reported (Cheniclet *et al.*, 2005), was devoted to analysing fruit development from anthesis to ripening. We report here new data about the development of each of the pericarp cell layers during fruit growth. In experiment 2, performed in 2015, ovaries from young flowers and fruit up to 4 DPA were analysed in order to characterize cellular events at the time of anthesis and fruit set. Floral stages up to anthesis were identified according to Brukhin *et al.* (2003).

### Cytological analyses

**Fig. 1.**
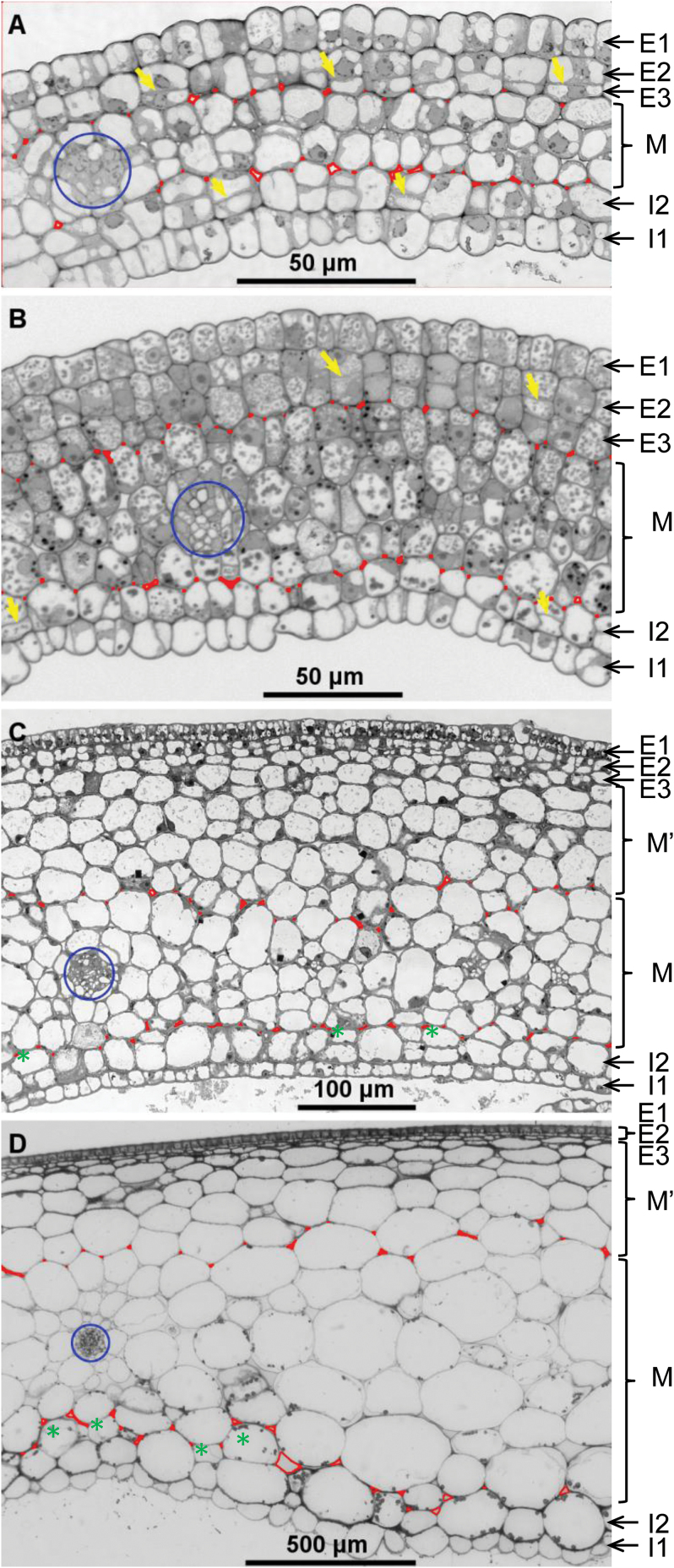
The structure of tomato pericarp during fruit growth. Tomato pericarp was observed on resin equatorial sections of ovaries at floral stages 11 (**A**) and anthesis (**B**), and of fruits at 4 DPA (**C**) and 36 DPA (**D**). Cell layers are named according to their relative positions: E1, outer epidermis; E2 and E3, two cell layers below E1; M, ~4 cell layers present at anthesis and surrounding vascular bundles (blue circles); I2, cell layer just close to I1, the inner epidermis. The M’ cell layers are formed after anthesis mostly from E2 and E3 but also, to a lesser, non-continuous extent from I2 (green asterisks, C, D). Yellow arrows show new cell walls indicative of recent periclinal cell divisions in E2 and I2 cell layers (A, B). Extracellular spaces at the border between M and adjacent layers E3, M’, or I2 are red. They are also present within M layers (not shown) but scarcely between the cells of other cell layers.

The pericarp has been divided into six groups of cell layers as shown in Fig. 1. The mean individual cell volume and the number of cells in each representative cell layer from a whole fruit were calculated as explained below. Notations used in these calculations are indicated in Table 1. The equatorial perimeter (*p*) of each ovary or fruit up to 4 DPA was measured on digital images of equatorial sections. For older fruits, it was calculated from the equatorial fruit diameter. In experiment 1, ovaries and fruits were prepared for resin section analysis as previously described (Cheniclet *et al.*, 2005). In experiment 2, the glutaraldehyde fixation was followed by post-fixation with 1% osmium tetroxide for 4 h at 4°C and the Technovit 7100 resin (Kulzer, Wehrheim, Germany) was replaced with Epon (Agar Scientific, Stansted, UK). Sections 1–2-µm thick were stained with toluidine blue and imaged on a Zeiss Axiophot microscope with a Spot RTKE camera (Diagnostic Instruments, Sterling Heights, MI, USA). Images were analysed with ImagePro-Plus software (Media Cybernetics, Silver Spring, MD, USA). The pericarp thickness and the number of cell layers across it were estimated in three pericarp portions devoid of vascular bundles.

**Table 1.** Notations for parameters used to calculate cell size and cell volume in given pericarp cell layers, and growth-associated parameters

Level	Notation	Meaning
Fruit	*p*	Fruit perimeter
	*t*	Pericarp thickness
	*L*	Number of cell layers across pericarp (*L*_0_ at anthesis)
Cell layer	*h*_*i*_	Mean anticlinal cell diameter (height) in one given pericarp cell layer *i*
	*w*_*i*_	Mean periclinal cell diameter (width) in one given pericarp cell layer *i*
	*A*_*i*_	Mean cell area in an equatorial pericarp section of one given cell layer *i*
	*V*_*i*_	Mean cell volume in one given pericarp cell layer *i*
	*N*_*i*_	Total cell number per fruit in one given pericarp cell layer *i*
Growth rate	*X*	Fruit volume, pericarp volume, cell number, or cell volume variation in whole pericarp or in a given cell layer
	*k*	The relative rate of fruit volume, pericarp volume, cell number, or cell volume variation, calculated as d*X*/(*X* × d*t*)
	*T*	The period for *X* to double its value, calculated as ln(2)/*k* during exponential growth

A group of cells from the outer epidermis (E1), the outer sub-epidermal (E2) and inner sub-epidermal (I2) cell layers, and the inner epidermis (I1) (Fig. 1) was manually delimited (see Supplementary Fig. S1A at *JXB* online) and its cell number, periclinal length, and area measured. For each fruit, these measurements were made in three pericarp portions, each representing 107 ± 48 cells per fruit according to the cell layer and to the developmental stage. These values were used to calculate the mean cell periclinal diameter (*w*_*i*_) and anticlinal diameter (*h*_*i*_) in each cell layer (*i*) of a given fruit (Supplementary Fig. S1A). The third cell dimension, the cell longitudinal diameter, was estimated as equal to *w*_*i*_ from control measurements in fruit longitudinal sections. Unless otherwise indicated, *V*_*i*_, the mean cell volume in a given cell layer *i*, was calculated by assuming a parallelepipedic shape for E1, E2, I2, and I1 cells:

$$V_{i}=w_{i}{}^{\text{2}}\times h_{i}$$ (1)

The number of cells in the E1 cell layer of the whole pericarp of one fruit, *N*_E1_, was calculated by assuming fruit shape as a sphere and by dividing the external area of this sphere by the mean periclinal area of one E1 cell (*w*_E1_^2^)

$$N_{\text{E1}}=\;(p^{\text{2}}/\pi )/w_{\text{E1}}{}^{\text{2}}$$ (2)

A similar method was used to calculate *N*_*i*_, the number of cells in each inner cell layer *i*. The fruit diameter was then decreased by twice the sum of the anticlinal diameters of all pericarp cell layers more external than cell layer *i*. The resulting diameter was used to calculate the surface of the sphere formed by the given cell layer *i*.

Because central mesocarp M cells may form non-continuous layers owing to the presence of vascular bundles within them, they were analysed by manually delimiting clusters of M cells from one to four cell layers, of which only the area and cell number were measured to calculate *A*_M_, the mean sectional area of one M cell. Up to 12 DPA, M cells do not have a preferential elongation axis in the cherry tomato line Wva106. They were then considered to have a cubic shape with a mean volume *V*_M_ = *A*_M_^3/2^. After 12 DPA, M cells show a preferential periclinal expansion, together with larger extracellular spaces. They were then considered as ellipsoid with their volume calculated as in Fanwoua *et al.* (2012):

$$V_{\text{M}}=\left(\text{2}/\text{3}\right)\times A_{\text{M}}\times h_{\text{M}}$$ (3)

where *h*_M_, their anticlinal diameter, was calculated as being 9% smaller than their periclinal diameter at 13 and 15 DPA, and 17%, 23%, 29%, and 33% smaller than their periclinal diameter at 17, 20, 29, and 36 DPA, respectively.

The cell size and cell number in the new M’ cell layers formed after anthesis were deduced from the previous measurements according to the following method. For each fruit, the number of M’ cell layers was calculated as the difference between the total number of cell layers in this fruit (*L*) and the mean number of cell layers at anthesis determined in other fruits (*L*_0_). The mean anticlinal diameter of one M’ cell (*h*_M’_) was then calculated from pericarp thickness *t* according to the formula:

$$h_{\text{M}’}=(t-h_{\text{E1}}-\text{2}\times h_{\text{E2}}-(L_{0}-\text{ 5})\;\times h_{\text{M}}-h_{\text{I2}}-h_{\text{I1}})/(L-L_{0})$$ (4)

where 2 × *h*_E2_ layers accounts for E2 and E3, and (*L*_0_ − 5) × *h*_M_ for the (*L*_0_ − 5) central mesocarp cell layers initially present at anthesis. The mean cell number in one M’ cell layer, *N*_M’_, was calculated by dividing the area of a sphere limited by the middle of M’ cell layers by the mean periclinal area of one M’ cell. Up to 12 DPA, M’ cells were estimated to have a parallelepipedic shape with a mean periclinal area equal to *h*_M’_^2^. Thus, their number in one M’ cell layer was calculated as:

$$N_{\text{M}’}=\pi \times {\left(p/\pi -\text{2}\times h_{\text{E1}}-\text{4}\times h_{\text{E2}}-(L-L_{0})\times h_{\text{M}’}\right)}^{\text{2}}/h_{\text{M}’}{}^{\text{2}}$$ (5)

After 12 DPA, the shape of M’ and I2 cells shifted towards periclinally elongated ellipsoids and their mean volumes were calculated as described above for M cells.

The total cell number in the pericarp was calculated by adding cell numbers from all pericarp cell layers. The mean cell volume in pericarp was calculated as the mean cell volume, cell number-weighted, from all pericarp cell layers.

### Mitosis analysis

A vibrating blade microtome Microm HM 650V (Microm Microtech, Brignais, France) was used to obtain 30 µm-thick tomato pericarp sections from fresh ovaries and fruit up to 4 DPA. These equatorial sections were made up in 50 mM 2-amino-2-(hydroxymethyl)-1,3-propanediol, 150 mM NaCl (pH 7) (TBS), fixed in ethanol:acetic acid (3:1) for 20 min, incubated for 5 min in 70% ethanol, rehydrated in ethanol baths (50%, 40%, 20%) for 5 min each, and then washed for 5 min in TBS. Cell wall staining was performed for 1 min in a 10 µg·ml^−1^ Calcofluor White M2R (Sigma-Aldrich, St Louis, MO, USA) solution in TBS and sections were briefly washed three times for 30 s in TBS. Sections were mounted and counterstained in Vectashield (Vector Laboratories, Burlingame, CA, USA) containing 1.5 µg·ml^−1^ DAPI for DNA staining. Images were acquired with a Nikon Eclipse 800 epifluorescence microscope equipped with a CoolSNAP HQ2 camera (Photometrics, Tucson, AZ, USA) and fluorescence was recovered with a band pass filter from 435 to 485 nm under an excitation wavelength of 340–380 nm with a ×20 objective.

Because tomato chromosomes are small, prophases could not be unambiguously identified and only mitotic figures beyond prophase were detected (Cong *et al.*, 2002) and counted using the ImageJ software (imagej.nih.gov/ij/) and the Cell Counter plugin. Mitotic indices were calculated as the ratio of mitotic nuclei to the total number of nuclei counted, at the level of the whole-pericarp level and of each cell layer. At each stage a minimum number of seven fruits were analysed, with ~1320 nuclei counted per fruit. The orientation of cell division planes was identified from the angle made by a line parallel to the outer or inner epidermis and either the equatorial plate axis (metaphase) or a line separating the two daughter nuclei (anaphase and telophase) (Supplementary Fig. S1D–F). Three classes of cell division were thus visually sorted: anticlinal (α angle close to 90°), periclinal (α angle close to 0°), and oblique (α angle with intermediate values, which were thereafter found to fall between 20 and 80°).

### Other measurements

Flow cytometry was used to determine nuclei ploidy profiles from whole ovaries and from pericarp of developing fruits as reported by Cheniclet *et al.* (2005). Ploidy histograms were quantitatively analysed with Flomax software (Partec GmbH, Görlitz, Germany), after manual treatment to exclude noise. When the ovaries of various species were analysed for their ploidy patterns at anthesis, 2C values were calibrated from literature data about DNA content and from ploidy patterns in young leaves.

Daily data from experiment 1 were used to calculate the relative rates of fruit and pericarp volume increase, of cell number variation, and of cell expansion in whole pericarp and in given cell layers. By referring to *X* for any of these growth parameters (Table 1), they vary over time according to an exponential function: *X* = *X*_0_ × e^*kt*^. *k* can be calculated as the relative rate of growth: *k* = d*X*/(*X* × d*t*), which was estimated from linear regression on three coupled values of the variable at time *t*−1, *t*, and *t*+1. Moreover, *T*, the time for the parameter *X* to double its value, was calculated as *T* = ln(2)/*k* (Webster and MacLeod, 1980; Granier and Tardieu, 1998).

## Results

### Growth characterization at fruit set

**Fig. 2.**
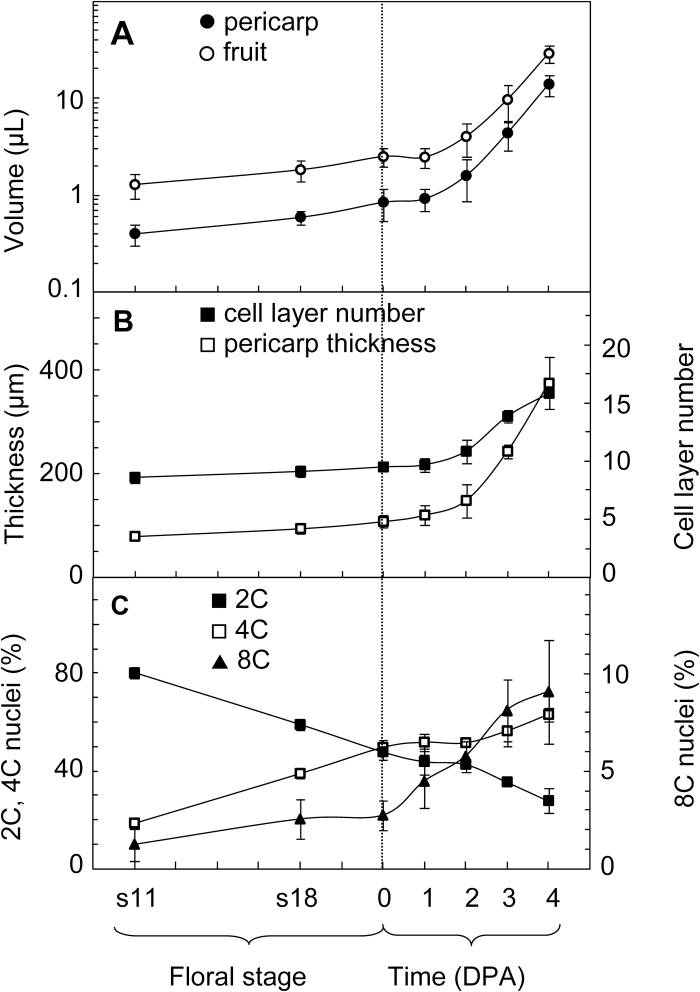
Fruit and pericarp growth at the time of fruit set. (**A**) Fruit volume and pericarp volume; (**B**) pericarp thickness and number of cell layers across the pericarp; (**C**) ploidy distribution within whole ovaries (up to 1 DPA) or dissected pericarp (2–4 DPA). Data are mean ± SD of measurements of 5.3 ± 1.8 ovaries or fruits in A and B, and of 4.0 ± 1.2 ovaries or fruits in C. The vertical dotted line marks the time of anthesis. The time scales for flower stages and for the post-anthesis period are not the same.

Mature ovaries are considered to undergo growth arrest in the days preceding pollination and fertilization. To appreciate the extent of this arrest, various growth-related variables were measured in the ovary and fruit of the cherry tomato Wva106 line at floral stages 11, 18, and anthesis, determined according to Brukhin *et al.* (2003), and up to 4 DPA. At stage 11, the young sepals are 4 mm long and meiosis starts in ovules. At stage 18, the corolla begins to open and becomes yellow, and the style stops elongating. In current conditions, ~7 and 2 days separated stage 11 and stage 18 from anthesis, respectively. We found that the tomato ovary displayed continuous growth from stage 11 to anthesis, as shown by a doubling of the whole ovary and pericarp volumes (Fig. 2A) and by a 25% increase in pericarp thickness (Fig. 2B). The number of cell layers in pericarp was nearly determined at stage 11, with one cell layer at most being added up to anthesis, when the pericarp has approximately nine cell layers (Fig. 1A, B; Fig. 2B). After anthesis, fruit and pericarp volumes, pericarp thickness, and the number of cell layers remained almost constant for 1 day. These four variables then increased more rapidly after 1 DPA, which indicates the success of fruit set and the early, vigorous growth of the new fruit (Fig. 2A, B).

2C, 4C, and 8C ploidy levels were detected in unpollinated ovaries as early as stage 11 (Fig. 2C). A continuous decrease of the frequency of 2C nuclei occurred from stage 11 (80%) to anthesis (49%). Reversely, the frequency of 4C nuclei increased during the same period from 19% to 49%. 8C nuclei were detected at a low frequency, <3%, in ovaries before anthesis. After anthesis, a rapid increase in 8C nuclei was observed as soon as the first DPA (Fig. 2C), and 8C nuclei amounted to 9% of pericarp nuclei at 4 DPA. Control experiments performed on ovaries or fruits dissected to remove central tissue and ovules/seeds nuclei confirmed that the same ploidy patterns occur in the ovary wall and pericarp.

The high level of 4C nuclei before anthesis may relate to G2 nuclei, or to a first endocycle. To address this question, the ploidy of ovary cells was compared at anthesis in various plant species which were chosen for the absence or occurrence of endoreduplication during fruit growth (see Supplementary Fig. S2 at *JXB* online). The eight species showing endoreduplication in their fruits displayed significantly smaller rates of 2C nuclei and larger rates of 4C nuclei than the six species without endoreduplication in their fruits. Moreover, 8C nuclei were detected in six species from the first group, but in none from the second group. High levels of 4C nuclei in ovaries at anthesis are thus correlated with traces of 8C nuclei and with the ability of the plant species to perform endoreduplication in fruits.

### Ontogeny of pericarp tissue through the ordered addition of cell layers

**Fig. 3.**
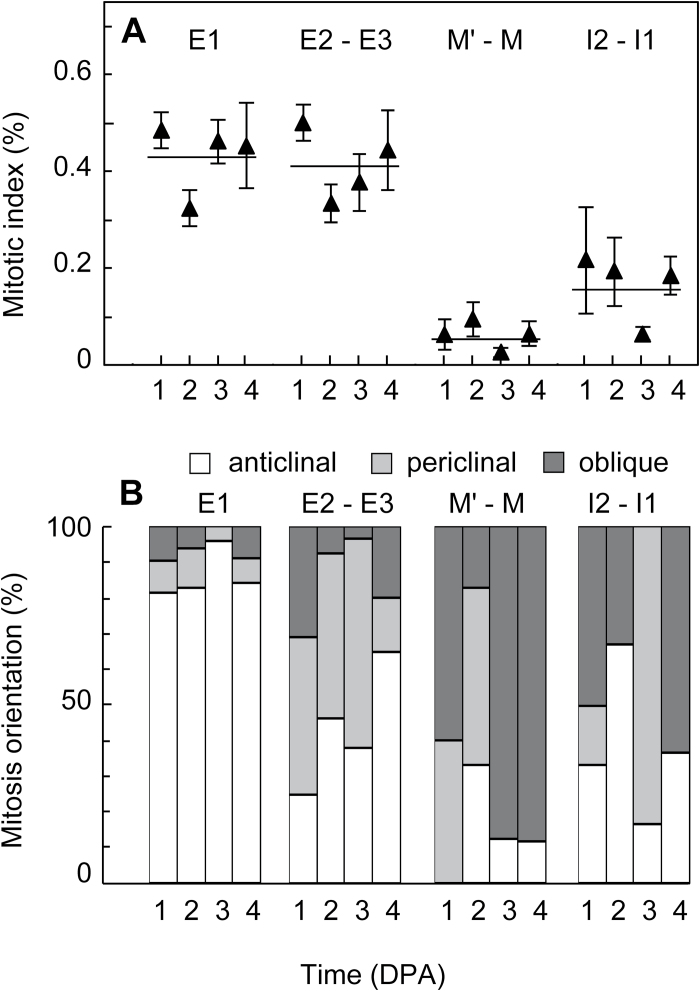
Mitotic activity in the tomato pericarp at fruit set. Mitoses were analysed in pericarp vibratome sections after DAPI staining at 1, 2, 3, and 4 DPA. Cell layers are named as in Fig. 1. A total number of 137 427 nuclei were analysed in the whole pericarp of 104 fruits, of which 30%, 19%, 36%, and 15% were located in E1, E2–E3, M’–M, and I2–I1 cell layers, respectively. (**A**) Mitotic indices (mean ± SE) including only metaphase, anaphase, and telophase figures were measured in each of the different cell layers of pericarp in seven fruits (1 and 2 DPA) or in 45 fruits (3 and 4 DPA). The horizontal line shows that there was no significant variation in mitotic index in a given cell layer within 1–4 DPA. (**B**) The orientation of cell division planes was determined from the pattern of mitotic nuclei as detailed in ‘Materials and methods’ (Supplementary Fig. S1B–F).

At anthesis the ovary wall is made up of nine cell layers which include both outer epidermis (E1) and inner epidermis (I1), two outer (E2 and E3) and one inner (I2) sub-epidermal cell layers, and four rows of central mesocarp cells (M) surrounding vascular bundles (Fig. 1B). No mitotic activity, as deduced from null mitotic indices, was detected in the ovary wall from flower stage 18 up to the time of anthesis. Mitotic activity resumed in the pericarp as soon as the first day after anthesis (Fig. 3A). It was highest in the three outer cell layers E1, E2, and E3, which displayed similar mitotic indices during the first 4 days after anthesis.

The orientation of cell division planes can be deduced from the observation of mitotic figures beyond prophase (Supplementary Fig. S1B). Anticlinal cell divisions, which increase the cell number in a given layer, were by far the most numerous in the pericarp (Fig. 3B), especially in the outer E1 epidermal cell layer, where their rate exceeded 80%. High levels of periclinal cell divisions were present only in E2 and E3 sub-epidermal cell layers (Fig. 3B, Supplementary Fig. S1B), with nearly 40% periclinal cell divisions from 1 to 3 DPA and a lower rate afterwards. These data strongly support the hypothesis that the new cell layers appearing after anthesis, indicated as M’ (Fig.1C, D; Supplementary Fig. S1), originate mostly from periclinal divisions in E2 and E3 cell layers. As a consequence, M’ cell layers were mostly located between E3 and M cell layers. However, lower mitotic activity with periclinal or oblique cell divisions also occurred in the I2 cell layer. This led to some M’ cells being located between I2 and M cell layers but not forming a continuous cell layer (see green asterisks in Fig. 1C, D).

The lowest mitotic activity in pericarp was found in M and M’ cell layers, mostly with periclinal or oblique cell divisions (Fig. 3A, B). No preferential orientation was noticed for oblique cell divisions in M’, M, and I2 cell layers. The four M cell layers present at anthesis in central mesocarp could be located at any stage of fruit growth from their position surrounding vascular bundles (Fig. 1). They showed the weakest, if any, mitotic activity among pericarp cell layers after anthesis. M cells differed from other pericarp cells because they displayed numerous intercellular spaces between them and at the border with neighbouring cell layers at all analysed stages of ovary and fruit growth. We have used this feature to identify M cell layers within the pericarp (Fig. 1, see red labels). By contrast, tight contacts without intercellular spaces were observed between E1, E2, and E3 cell layers, between I1 and I2 cell layers, and within M’ cells layers nearly up to the end of fruit growth.

### Time-course analysis of pericarp growth according to cell division and to cell expansion in each cell layer

**Fig. 4.**
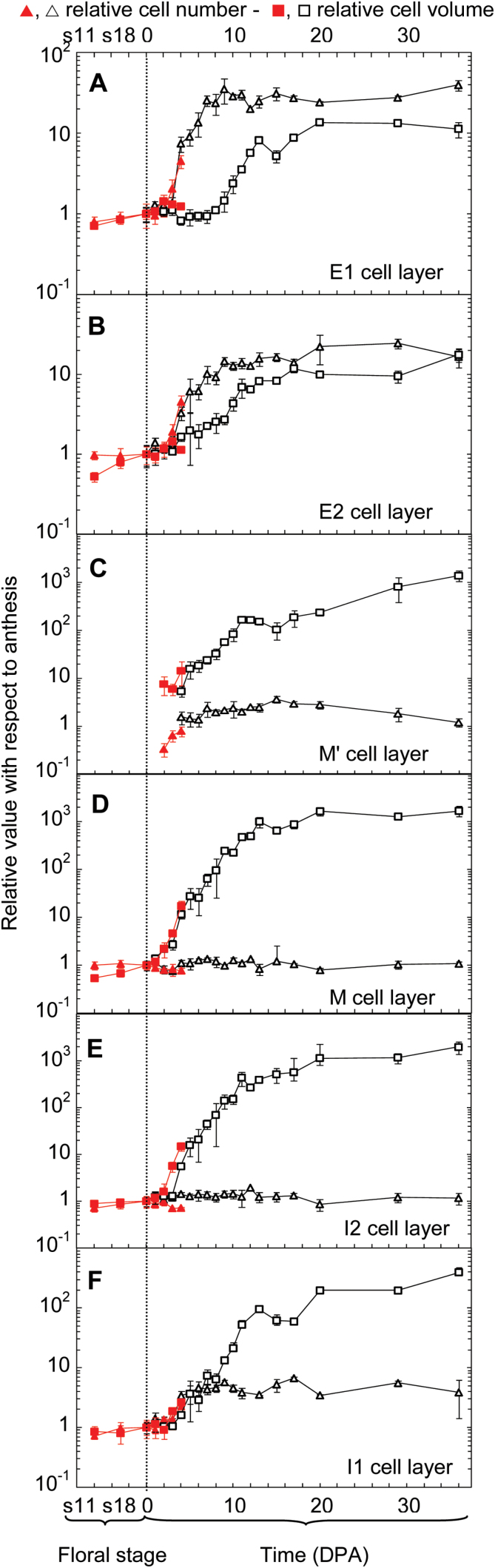
Time-course of cell number and cell volume in various cell layers of tomato pericarp during fruit growth. Each panel shows the evolution of cell number and of cell volume in one given cell layer. (**A**) E1 cell layer; (**B**) E2 cell layer; (**C**) M’ cell layer; (**D**) M cell layer; (**E**) I2 cell layer; (**F**) I1 cell layer. Variables are expressed as relative values with reference to the value of 1 at anthesis, indicated by the vertical dotted line. Two independent experiments are shown, first from anthesis to 36 DPA (dark lines and symbols), and second from flower bud stage 11 to 4 DPA (red lines and symbols). In current conditions, ~7 and 2 days separated stage 11 and stage 18 from anthesis, respectively. For the M’ cell layer (C), which results from periclinal cell divisions from E2 cells, the reference values are the values of the E2 cell layer at anthesis, and the variables are indicated from 4 and 2 DPA onwards in experiments 1 and 2, respectively. The data are mean ± SD for 3.5 ± 1.0 fruits in experiment 1 and 5.3 ± 1.8 fruits in experiment 2. The time scale is different for flower stages and DPA.

The whole cell number and the mean cell unitary volume of each pericarp cell layer were measured at daily intervals in two independent experiments, first from anthesis to 36 DPA (breaker stage) and second from flower bud stage 11 to 4 DPA. The results, expressed as relative values with reference to anthesis, showed that each cell layer displayed a specific pattern of cell number variation and cell expansion (Fig. 4).

The number and size of E1 cells increased slightly, by ~25%, between flower stage 11 and anthesis (Fig. 4A). Then, after a 2-day lag following pollination, E1 cell number increased by 30-fold up to 9 DPA with no change afterwards. Being nearly isodiametric at anthesis, E1 cells displayed a decrease in their periclinal diameter at 4 and 5 DPA (see Supplementary Fig. S3A at *JXB* online), likely because of ongoing anticlinal divisions. As their anticlinal diameter increased, their mean volume remained constant from 0 to 7 DPA before expanding by 12-fold from 8 to 20 DPA (Fig. 4A). After 11–12 DPA, these cells developed thick, cutinized walls and a cuticle (Segado *et al.*, 2016), which slowed down and then stopped any further expansion.

The number of E2 cells increased at the same time as E1 cells, but only by 19-fold (Fig. 4B). By contrast with E1 cells, the expansion of E2 cells started early, at 4 DPA, while their number was still increasing. They were isodiametric at anthesis, and their expansion occurred only on a periclinal axis (Supplementary Fig. S3B). The E3 cell layer just beneath E2 cells developed from periclinal divisions in E2 cells before flower stage 11, and it was completed between flower stage 11 and stage 18 (Fig. 1A, B). The characteristics of E3 cells are not shown here because they were quite close to those of E2 cells.

The central mesocarp M cells displayed a growth pattern very different from E1 and E2 cells (Fig. 4D). The mean cell number in one M cell layer remained unchanged from flower stage 11 to 36 DPA. However, M cells continuously expanded, first slightly before anthesis and then dramatically, by 1550-fold, from fruit set up to 20 DPA, with nearly no more expansion afterwards. M cells did not show any axis of preferential expansion in this tomato line up to 12 DPA (Fig. 1A–C). Then, they slightly elongated along a periclinal axis, with an ellipsoid shape and larger extracellular spaces (Fig. 1D). The pattern of cell number variation and cell expansion of I2 cells resembled that of M cells, with nearly no increase in cell number and a huge cell expansion by 1300-fold (Fig. 4E, Supplementary Fig. S3C).

The inner epidermis I1 showed an increase in cell number and cell volume, as for E1 and E2 cells (Fig. 4F). But I1 cells displayed a comparatively lower increase in cell number by only 4-fold from 3 to 9 DPA, and a larger increase in cell volume by 400-fold from 3 to 36 DPA. I1 cells increased their number and their volume at the same time up to 9 DPA, and they remained nearly isodiametric up to 12 DPA (Supplementary Fig. S3D). Their periclinal diameter then increased by ~50% more than their anticlinal diameter as for M, M’, and I2 cells.

The mean cell number in one single M’ cell layer increased by only 2.8-fold up to 9 DPA, as compared with cell number in one E2 cell layer at anthesis (Fig. 4C). Conversely, M’ cells displayed a 1350-fold increase in their volume. By contrast with M cells, which stopped expanding after 20 DPA, M’ cells kept on growing up to 36 DPA (Fig. 4C). Because of the rapid production of six to seven new cell layers shortly after fruit set, the whole number of M’ cells increased rapidly by 9-fold from 2 to 5 DPA, and up to 18-fold at 9 DPA (see Supplementary Fig. S4 at *JXB* online).

**Fig. 5.**
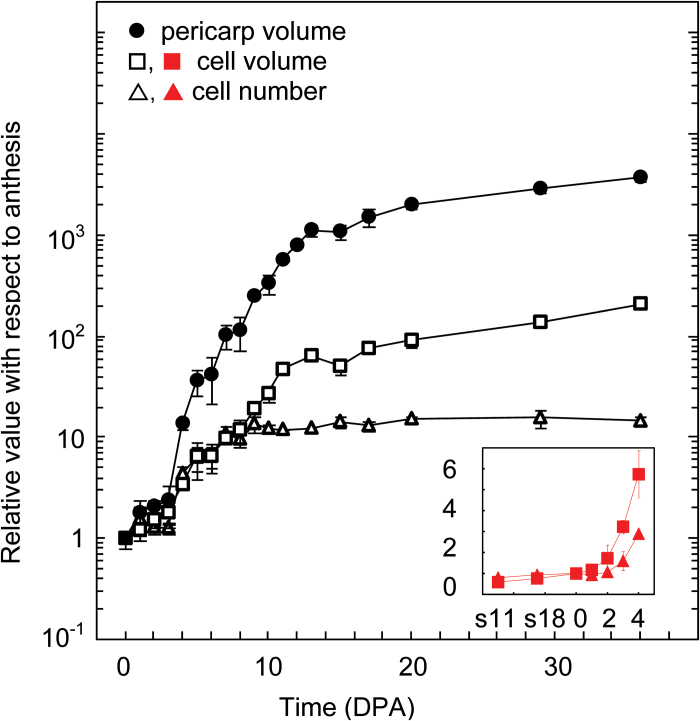
Cell multiplication and cell expansion during pericarp growth. The main panel shows data from experiment 1 in Fig. 4. The data show the mean ± SD values of pericarp volume, total cell number in pericarp, and cell volume according to fruit age. They are expressed as relative values with reference to the value of 1 at anthesis. The inset shows relative values ± SD of cell number and cell volume in experiment 2 from Fig. 4 for floral stages 11 up to 4 DPA. The following absolute values were found at anthesis in experiment 1 and 2, respectively: whole pericarp volume 0.539 ± 0.046 µL and 0.849 ± 0.311 µL; total cell number 0.411 ± 0.085 × 10^6^ and 0.562 ± 0.113 × 10^6^; mean cell volume 1.36 ± 0.30 pL and 1.49 ± 0.33 pL. This figure can be found in colour at *JXB* online.

**Fig. 6.**
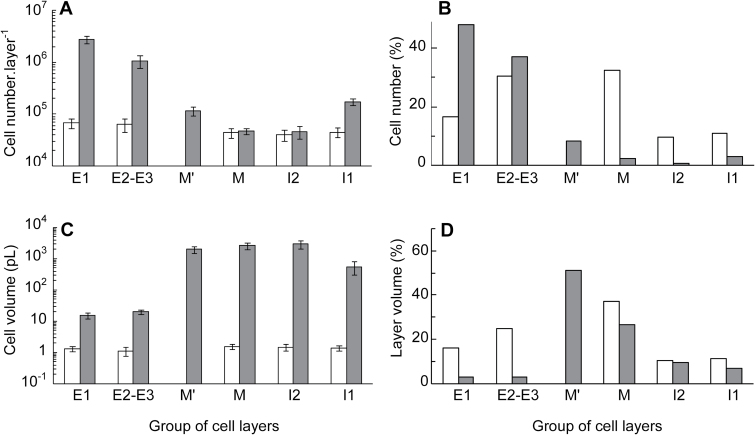
Characteristics of the different groups of cell layers in tomato pericarp at the beginning and the end of fruit growth. The data are from experiment 1 in Fig. 4 and show values at 0 DPA (white bars) and at 36 DPA (grey bars). (**A**) Cell number in each single cell layer (mean ± SD). (**B**) Relative value of cell number in each group of cell layers with reference to the total pericarp cell number. (**C**) Mean cell volume ± SD in each group of cell layers. (**D**) Relative volume occupied by each group of cell layers in the pericarp. At anthesis and at 36 DPA, there was one cell layer in groups E1, I2, and I1, two cell layers in group E2–E3, and four cell layers in group M. There was 0 cell layers in group M’ at anthesis and 6.4 layers at 36 DPA.

The overall dynamics of cell number and of mean cell size during tomato pericarp growth are shown in Fig. 5. Both variables started to increase at 2 DPA, corresponding to fruit set. The total cell number in pericarp increased by 15-fold within 36 DPA and most of this increase occurred exponentially within 9 DPA. The mean pericarp cell volume, excluding vascular bundles, increased by 170-fold up to 36 DPA, and most of this increase occurred exponentially within 14 DPA. The absolute values of cell numbers and cell volume in each single cell layer at anthesis and at 36 DPA are shown in Fig. 6A, C, which illustrates the opposite gradients of cell division and cell expansion from the outermost E1 to the innermost I1 cell layer. The relative cell number and whole volume of each group of cell layers within pericarp are shown in Fig. 6B, D.

**Fig. 7.**
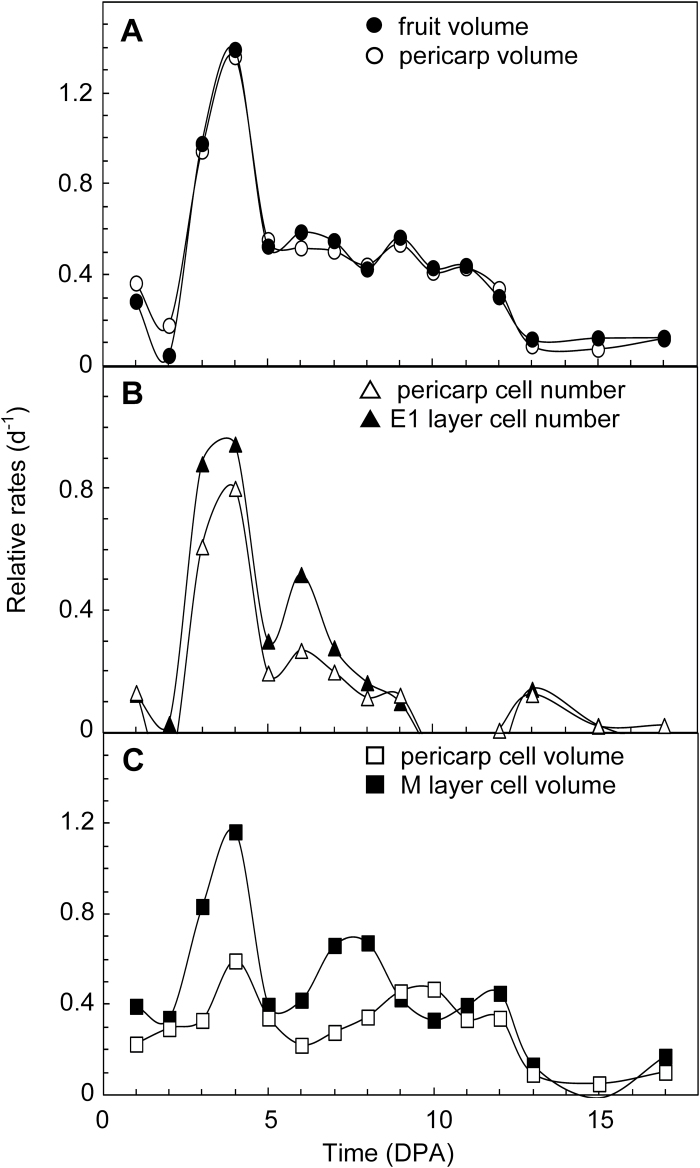
Relative rates of fruit and pericarp growth, cell production, and cell volume increase in tomato. The data are from experiment 1 reported in Figs 4 and 5. Each point represents the slope of ln(variable) over three successive daily time points. (**A**) Relative rates of fruit and pericarp growth in volume. (**B**) Relative rates of cell production in pericarp and in E1 cell layer. (**C**) Relative rates of cell expansion in volume in pericarp and in the M cell layer.

These data were used to calculate several relative growth rates during tomato fruit development (Fig. 7). The relative rates of fruit and pericarp increase in volume were strikingly similar to each other throughout fruit development, with a clear maximum of 1.4 µL·µL^−1^·day^−1^, that is, 1.4 day^−1^ at day 4 (Fig. 7A). The relative rate of cell production in pericarp also reached a maximum value of 0.80 cell·cell^−1^·day^−1^, that is, 0.80 day^−1^ at day 4 (Fig. 7B). When only the E1 or the E2 cell layer was considered, the maximum relative rates of cell production were 0.95 day^−1^ or 0.75 day^−1^ at day 4, respectively (Fig. 7B; the curve for E2 cells is not shown but nearly superimposed on that for all pericarp cells). The maximum value of the relative rate of cell expansion was also reached at day 4, being 0.63 pL·pL^−1^·day^−1^, that is, 0.63 day^−1^ at the level of the whole pericarp and 1.16 day^−1^ at the level of the central mesocarp M cells (Fig. 7C).

## Discussion

### Cell divisions resume rapidly in specific cell layers, with an estimated cell-cycle duration close to 14 h

This study reports for the first time detailed quantitative data about cell numbers in tomato pericarp throughout fruit growth and at the cell-layer level. Post-anthesis cell divisions resume as soon as 1 DPA and they are mostly located in the three outer pericarp cell layers and, to a lesser extent, the two inner pericarp cell layers (Fig. 3A, Fig. 4), as previously reported qualitatively (Gillaspy *et al.*, 1993; Xiao *et al.*, 2009; Pabón-Mora and Litt, 2011). Cell divisions extend up to 9 DPA, as in other small-fruited tomato lines (Gillaspy *et al.*, 1993; Xiao *et al.*, 2009; Pabón-Mora and Litt, 2011). Longer durations of the cell division phase have been reported in tomato lines with larger fruits (Bohner and Bangerth, 1988; Cong *et al.*, 2002; Bertin *et al.*, 2009). The total cell number in the pericarp of one ovary at anthesis increases by 15-fold in fully grown fruit, a value similar to the one reported by Teitel *et al.* (1985), but larger than the few other values previously reported in various tomato lines, even with larger fruits (Bünger-Kibler and Bangerth, 1982; Bertin *et al.*, 2009; Fanwoua *et al.*, 2012). Underestimation of cell numbers in tomato pericarp from fully grown fruits may be related to an underestimation of cell numbers in the three outer pericarp cell layers. We show here that these layers contain 85% of pericarp cells whereas they account for only 6% of the pericarp volume because of the small unitary volume of their cells (Fig. 6C, D).

The maximum relative rate of cell production in pericarp occurs in E1 and E2 cell layers at 4 DPA (Fig. 7B). The values reported in the result section only include anticlinal cell divisions because cells were numbered in one given cell layer. After correction for 15% of E1 cells and 35% of E2 cells undergoing mitosis according to a non-anticlinal division plane at 4 DPA (Fig. 3B), the maximum relative rates of cell production shift to 1.12 day^−1^ for E1 cells and 1.15 day^−1^ for E2 cells. With the assumption that all of these cells are actively cell cycling in an asynchronous way (Webster and MacLeod, 1980), these data correspond to a cell-cycle duration of 14.9 h and 14.4 h for E1 and E2 cells, respectively. Larger values from 24 h to 62 h (Bertin *et al.* 2003*a*, 2007) were deduced from a whole-pericarp cell population during the entire fruit-growth period. They thus include cycling and non-cycling cells from all pericarp cell layers. The values reported here are much closer to the cell-cycle duration of 10.6 h reported in tomato root tip from ^3^H-thymidine incorporation (Van’t Hof, 1965). They reflect very active cell multiplication in the outer cell layers of tomato pericarp at the onset of fruit growth. Very few values of cell-cycle duration have been reported in tomato and more generally in plant cells, although this parameter is critical for unravelling and modelling organ growth.

### The onset of new cell layers after anthesis is specifically regulated by periclinal cell divisions in the two outer sub-epidermal layers

The number of cell layers across the ovary wall is nearly achieved by flower stage 11, in agreement with previous data in tomato and related species (Mazzucato *et al.*, 1998; Xiao *et al.*, 2009; Pabón-Mora and Litt, 2011). The observation, prior to anthesis, of numerous thin periclinal cell walls between E2 and E3 cells (Fig. 1A, B) provides strong support to the fact that the E3 cell layer is the last cell layer created before anthesis in the cherry tomato line Wva106, and that it originates from periclinal E2 cell divisions.

A strikingly constant number of cell layers, nine to 12, was found at anthesis in 20 very different tomato lines (Cheniclet *et al.*, 2005) as well as in other lines (Roth, 1977; Mazzucato *et al.*, 1998; Cong *et al.*, 2002; Pabón-Mora and Litt, 2011; Fanwoua *et al.*, 2012). By contrast, the number of new cell layers formed after anthesis is highly variable, from, for example, five to 25 in 20 tomato lines (Cheniclet *et al.*, 2005). Most of the new cell layers are formed early in the Wva106 line pericarp, between 3 and 5 DPA, as also shown in *S. pimpinellifolium*, another cherry tomato (Xiao *et al.*, 2009), whereas the cell division period extends up to 9 DPA. This suggests a differential regulation between periclinal, cell layer-forming cell divisions and the bulk of other cell divisions. Such a differential regulation between the two types of cell divisions is also suggested from the differential effect of temperature (Fanwoua *et al.*, 2012) and of genetic determinants (Cong *et al.*, 2002) on these two types of cell divisions.

### Dramatic cell expansion occurs as soon as fruit set, with a cell volume doubling time of 14 h

At anthesis, all pericarp cells, apart from vascular bundles not included in this study, have roughly the same volume, ~1.6 pL (Fig. 6C). Cell expansion starts as soon as 2 DPA and occurs in a highly differential way according to cell layers. It is by far the most important in central mesocarp M cells, as also reported in *S. pimpinellifolium* (Xiao *et al.*, 2009). Early cell expansion in tomato pericarp at 1–2 DPA has also been deduced from the observation that most pericarp cells display enlargement of small vacuoles to form a large central vacuole at that time (Mohr and Stein, 1969).

Even if the absolute rate of volume increases in cells and fruit are largest between 10 and 20 DPA, their relative rates are maximized at 4 DPA, as previously shown for cell production (Fig. 7). M cells, which contribute to growth only by cell expansion, not by cell division, show a maximum relative rate of cell expansion of 1.16 day^−1^, which corresponds to a mean doubling time of cell volume of 14.3 h, that is, identical to the estimated length of the cell cycle in E1 and E2 cell layers. This suggests that the fast fruit growth just after fruit set is contributed to equally by cell division and by cell expansion in specific cell layers. It will be of interest to study the effect on these cell parameters of genetic and environmental factors which modify the balance between cell division and cell expansion.

At the whole-pericarp level, cell division and cell expansion occur at the same rate up to 9 DPA (Fig. 5). However, as cell expansion lasts longer, it has a larger effect than cell division on pericarp growth, as previously reported for *Solanaceae* fleshy fruits (Pabón-Mora and Litt, 2011). As a whole, the 2600-fold increase in pericarp volume up to 36 DPA relies on a 15-fold increase in cell number and a 170-fold increase in cell volume. Both processes contribute significantly to pericarp and fruit growth, and their combination supports the dramatic increase in pericarp and fruit volume.

The final growth of each cell layer was then calculated as the product of cell number and cell volume rates of change between anthesis and 36 DPA. The three outer cell layers grew by 230- to 390-fold each, whereas the more inner layers grew by 1200- to 1900-fold each. It is remarkable that these two sets of data fall in the range of the increase in fruit outer area (×230) and in pericarp and fruit volumes (approximately ×3000, with 50% of growth due to new M’ cell layers, Fig. 6D), respectively. The growth of the three outer cell layers is mostly periclinal and restricted to accompanying the increase in fruit surface. Reversely, the growth of the more inner cell layers contributes significantly to the increase in fruit volume by anticlinal and periclinal expansion. The difference in growth and fate between outer and inner cell layers reported here provides support to a specific role for outer cell layers in tomato fruit growth (Thompson *et al.*, 1998; Thompson, 2001).

### Endoreduplication starts before anthesis and may account for the absence of cell division and the rapid expansion of central mesocarp cells

Previous studies reported high 4C levels among pericarp cell nuclei at anthesis (Bertin *et al.*, 2003*b*; Cheniclet *et al.*, 2005; Bertin *et al.*, 2007). We here confirm these data and lend complementary support to the hypothesis that endoreduplication is initiated in tomato ovary before anthesis. Cycling G2-related 4C nuclei should not be at a high level from flower stage 18 to anthesis because we did not detect any mitotic activity in the tomato ovary wall at these stages, in agreement with previous data (Gillaspy *et al.*, 1993; Tanksley, 2004; Xiao *et al.*, 2009; Pabón-Mora and Litt, 2011). Moreover, we report that high rates of 4C nuclei (25–50%) are present in the mature ovaries of plant species displaying endoreduplication but not in the ones without endoreduplication in their fruits (Supplementary Fig. S2). Thus, the majority of 4C nuclei in ovary walls from flower stage 18 to anthesis originate from the first endocycle and must be considered as polyploid. The presence of 8C nuclei in the ovary wall before anthesis, and only in fruit species displaying endoreduplication (Fig 2C, Supplementary Fig. S2), demonstrates that a second endocycle occurs before anthesis. Thus, endoreduplication starts in young tomato ovaries at the end of organogenesis-related cell divisions. A similar situation has been reported in Arabidopsis leaf primordia (Beemster *et al.*, 2005).

At the end of fruit growth, most pericarp cells are polyploid, and the largest ploidy levels, with six to seven endocycles, have been located exactly in central mesocarp M cells (Bourdon *et al.*, 2011). From the hypothesis that the largest ploidy levels would be reached in cells that start endocycling soonest, it is likely that the central mesocarp M cells are the first cells to become polyploid in young ovaries. Our data suggest that they all are at least 4C at anthesis, and that some are already 8C.

Unlike endomitosis, which increases chromosome number and does not preclude further mitosis, endoreduplication in tomato fruit relies on polyteny (Bourdon *et al.*, 2011) and further mitoses are not considered to occur in this case, although some exceptions have been reported (Bradley and Crane, 1955; Roth, 1977). The observation that the gradient of cell production in pericarp after anthesis (Fig. 6A) is exactly opposite to the pattern of ploidy in this tissue (Bourdon *et al.*, 2011) supports this hypothesis. We report here for the first time a rapid increase in 8C nuclei just after anthesis, at 1 DPA. The early shift of central mesocarp M cells towards further endocycles could thus explain the absence of a regular cell cycle in these cell layers after anthesis.

A second consequence of the early increase in ploidy of M cells may be their dramatic expansion from 2 DPA onwards. A significant negative correlation was found between endoreduplication and the duration of fruit growth in several fruit species (Chevalier *et al.*, 2011; Pirrello *et al.*, 2014), which suggests that, in addition to its potential effect on cell and organ size, endoreduplication may also provide the ecological benefit of faster organ growth (Barow, 2006; De Veylder *et al.*, 2011; Scholes and Paige, 2015). Our results strengthen the hypothesis that early endoreduplication in mesocarp cells supports their very rapid cell expansion after pollination and fertilization.

### The cell layer-based analysis of tomato pericarp development helps in the terminology for pericarp patterning

The terms exocarp, mesocarp, and endocarp are widely used to designate specific regions in pericarp. However, they are applied inconsistently according to authors, even in the single tomato fruit species (Roth, 1977; Pabón-Mora and Litt, 2011; Seymour *et al.*, 2013). The study of pericarp development at the cell-layer level provides some argument to clarify these terms in the case of tomato.

The three outer cell layers in tomato pericarp, or slightly more in larger tomato lines, form the exocarp (E1, E2, and E3 in this report). They display the largest mitotic activity among pericarp cells after anthesis, with specifically oriented cell divisions, low cell expansion, and a low ploidy level (Bourdon *et al.*, 2011). Their overall growth has the same magnitude as the increase in the outer area of the fruit.

In a symmetrical way, the inner epidermis (I1) and sub-epidermis cell layers (I2) represent the endocarp. As in the exocarp, these two cell layers divide according to specifically oriented planes. Endocarp cells have a lower mitotic activity, a larger ploidy level, and a larger rate of expansion than exocarp cells. Their overall growth has the same magnitude as the increase in fruit and pericarp volume.

The central mesocarp cells already present at anthesis and surrounding vascular bundles (M), together with the new cell layers formed after anthesis (M’), constitute the mesocarp. M’ cells mostly originate from the exocarp but also to some extent from the endocarp. In tomato, the mesocarp is characterized by null or very weak, not specifically oriented cell divisions, huge cell expansion, and a high ploidy level, with M cells being still more polyploid than M’ cells (Bourdon *et al.*, 2011). Its growth has the same magnitude as the increase in fruit and pericarp volume.

### The same hormonal signals could activate cell division, cell endoreduplication, and cell expansion during fruit set

One major result of the current study is that the earliest cellular response to fruit-inducing pollination and fertilization is the simultaneous increase at 1 DPA in mitotic index in exocarp and in 8C nuclei likely in the central mesocarp. This suggests a common regulatory signal for these two responses. Auxin and gibberellins have been identified as early signals for fruit set and vigorous fruit growth, including tomato (Pattison *et al.*, 2014; Sotelo-Silveira *et al.*, 2014). Both hormones display positive effects on cell division and cell expansion (Sablowski and Carnier Dornelas, 2014; Takatsuka and Umeda, 2014). A decrease in auxin signalling as well as gibberellin stimulation have been shown to trigger the shift from a regular cell cycle to the endocycle (De Veylder *et al.*, 2011). We propose that these two hormones could simultaneously activate the cell cycle and the endocycle in different cell layers according to absolute levels, cell sensitivity, and other signalling cross talks.

The identification and quantitative analysis of a cell layer-associated specific pattern of cell division and cell expansion during tomato fruit development provides a detailed framework that will contribute to further phenotyping and modelling of tomato fruit growth. Future work would aim to decipher the impact of environmental cues on cellular responses during fruit growth in relation to hormone signalling. The results of the current study will thus reinforce the systems biology of fruit set and fruit growth in plants.

## Supplementary data

Supplementary data are available at *JXB* online.

Fig. S1. Measurement of cell dimension and of mitotic activity in tomato pericarp

Fig. S2. Ploidy levels in ovaries of various fruit species

Fig. S3. Time-course of pericarp cell dimensions during pericarp growth

Fig. S4. Characterization of M’ cell layers

## Supplementary Material

Supplementary Figures S1-S4
