# Supplementary material for: Cell layer-specific patterns of cell division and cell expansion during fruit set and fruit growth in tomato pericarp
Source: J Exp Bot. 2017 Mar 28;68(7):1613–23. doi: 10.1093/jxb/erx058 (PMC5444452; doi:10.1093/jxb/erx058)
Supplement: Supplementary Figures S1-S4 [file erx058_suppl_Supplementary_Figures_S1-S4.docx]

**Title :**

Cell-layer specific patterns of cell division and cell expansion during fruit set and fruit growth in tomato pericarp

**Authors names**

Jean-Pierre Renaudin, Cynthia Deluche, Catherine Cheniclet, Christian Chevalier, Nathalie Frangne

**Supporting Information**

**Fig. S1.** Measurement of cell dimensions and of mitotic activity in tomato pericarp. (A) Bright-field image of the outer part of a 3 DPA pericarp Epon section. The cell layers are named according to Fig. 1. The measurement of cell size is illustrated for the epidermal E1 cell layer. A box containing all E1 cells in a pericarp portion is manually drawn (black line). For clarity, the box contains only a limited number of E1 cells. The length (dotted line) of this box and the number of E1 cells enclosed were used to estimate the mean periclinal cell diameter (w). The mean cell anticlinal diameter (h) was estimated as the ratio of the boxed area to w and to the cell number in this area. (B, C) Epifluorescence images of 30-40 µm vibrating microtome sections of a 4 DPA pericarp showing DAPI-stained nuclei and Calcofluor White M2R-stained cell walls. In (B), two mitotic figures at the anaphase-telophase stage are present in E1 cell layer (a, anticlinal orientation of the cell division plane) and in E2 cell layer (p, periclinal orientation of the cell division plane). In (C) an oblique cell division is indicated (o) in a M' cell layer. (D,E,F) Enlargement of mitotic nuclei displayed in (B,C). The plain white line is parallel to outer epidermis, while the dotted white line figures the mitotic division plane. The sorting of mitotic figures as periclinal, anticlinal or oblique is visually made from the angle α.

**Fig. S2.** Ploidy levels in ovaries of various fruit species. Flowers at anthesis from 14 species were harvested and the ploidy levels of their ovaries were assayed by flow cytometry. The white bars show the mean ± SD for the 6 species in the fruit of which no endoreduplication occurs during fruit development : dipladenia, jasmine, kiwi, oleander, pansy and petunia. The grey bars show the mean ± SD for the 8 species in the fruit of which significant endoreduplication occurs during fruit development : apricot, bean, cherry, cucumber, pepper, tobacco, tomato and zucchini. Asterisks indicate significant differences (P=0.95) between the two groups of species.

**Fig. S3.** Time-course of pericarp cell dimensions during pericarp growth. The data are from experiment 1 in Fig. 4. They show the mean ± SD of the cell periclinal diameter (dark squares) and of the cell anticlinal diameter (open squares) in cell layers E1 (A), E2 (B), I2 (C) and I1 (D).

## Fig. S4. Characterization of M’ cell layers. Data from experiments 1 (dark lines, dark symbols) and 2 (red lines, red symbols) show mean ± SD of the number of M’ cell layers (A) and of the total number of M' cells (B) from 0 DPA to 36 DPA. The latter variable is expressed with reference to the number of cells in one E2 cell layer at anthesis. In (B), M' cell numbers are indicated from 4 DPA and from 2 DPA for experiment 1 and 2, respectively.

Figure S1

Figure S2

Figure S3




Figure S4
